# The role of kinship and demography in shaping cooperation amongst male lions

**DOI:** 10.1038/s41598-020-74247-x

**Published:** 2020-10-16

**Authors:** Stotra Chakrabarti, Vishnupriya Kolipakam, Joseph K. Bump, Yadvendradev V. Jhala

**Affiliations:** 1 Department of Fisheries, Wildlife & Conservation Biology, University of Minnesota, 2003 Buford Circle, 150 Skok Hall, St. Paul, MN 55108 USA; 2 Department of Animal Ecology & Conservation Biology, Wildlife Institute of India, Chandrabani, Dehradun, Uttarakhand 248 001 India

**Keywords:** Behavioural ecology, Ecological genetics, Evolutionary ecology

## Abstract

The influence of kinship on animal cooperation is often unclear. Cooperating Asiatic lion coalitions are linearly hierarchical; male partners appropriate resources disproportionately. To investigate how kinship affect coalitionary dynamics, we combined microsatellite based genetic inferences with long-term genealogical records to measure relatedness between coalition partners of free-ranging lions in Gir, India. Large coalitions had higher likelihood of having sibling partners, while pairs were primarily unrelated. Fitness computations incorporating genetic relatedness revealed that low-ranking males in large coalitions were typically related to the dominant males and had fitness indices higher than single males, contrary to the previous understanding of this system based on indices derived from behavioural metrics alone. This demonstrates the indirect benefits to (related) males in large coalitions. Dominant males were found to ‘lose less’ if they lost mating opportunities to related partners versus unrelated males. From observations on territorial conflicts we show that while unrelated males cooperate, kin-selected benefits are *ultimately* essential for the maintenance of large coalitions. Although large coalitions maximised fitness as a group, demographic parameters limited their prevalence by restricting kin availability. Such demographic and behavioural constraints condition two-male coalitions to be the most attainable compromise for Gir lions.

## Introduction

Cooperation amongst individuals is a puzzling facet of sociality because it often entails different immediate fitness outcomes for actors and recipients^1^. But for cooperation to attain stability, both actors and recipients should benefit from such acts over the long term. Benefits for a cooperating individual are based on fitness accrued through personal acts (direct effects) and/or by aiding related individuals (indirect effects)^1^. However, cooperation generally induces differences in the magnitudes of direct and indirect effects between individuals of a group, wherein inclusive fitness of certain individuals is primarily due to direct effects while for other individuals, indirect benefits predominate (please refer to Glossary for a definition of key terms used throughout the paper). For example, in hierarchical societies with few breeding individuals, the *breeders* appropriate direct fitness by producing offspring while non-breeding *helpers* boost their inclusive fitness indirectly by helping related breeders and their offspring^1, 2^. Since unit effect of direct and indirect benefits are not equivalent, an individual’s decision to cooperate is based on the cost-benefit trade-offs that originate from such discrepancies over the long-term^2,3^. These trade-offs are primarily resource and socially mediated, and consequently such decisions are context driven and often exhibit plasticity across temporal and spatial scales^4,5^, resulting in diverse instances and pathways of cooperation. For example, in common vampire bats *Desmodus rotundus* food-sharing is better explained by previous mutualistic encounters than relatedness between the donors and recipients^6^. While mutualism serves as the base for certain societies, in some other species cooperation is typically or ‘nepotistically’ restricted to kin, such as in carrion crows *Corvus corone*^7^, spotted hyenas *Crocuta crocuta*^8,9^, Seychelles warblers *Acrocephalus sechellensis* (reviewed in Ref.^10^) and male coalitions of wild turkeys *Meleagris galapavo*^11^. Thus, although the tendency to cooperate has been primarily ascribed to fundamental mechanisms like mutualism and kin-selection, the combination and magnitude of these drivers vary among systems. Furthermore, although kin support provides a theoretical framework for how direct and indirect fitness effects can stabilize group formation, such theory is often constrained in real life by the availability of related individuals (reviewed in Ref.^12^).

Male-male cooperation to form coalitions are ideal models to comprehend these drivers that affect individual decisions to cooperate^13^ because coalition formation in males is not the general norm. Rather, natural and sexual selection results in males competing with each other for access to resources (such as food, mates and territories), while coalitionary tendency often makes them share the same^14^.

Male coalitions have been reported from several species^13^, but have been studied in great detail in lions *Panthera leo*^15–20^. Male lions team up to gain access to females and their territories, to harness breeding and foraging benefits. A typical lion coalition comprises of 2–9 adult males that function as a unit, guarding group(s) of lionesses from other coalitions^15,20,21^. Larger coalitions have greater competitive prowess and consequently individuals in such coalitions generally have higher lifetime success compared to males in small coalitions^17,18^. Although male partners in a coalition cooperate and function as a unit, competition between partners can be intense. This competition is somewhat relaxed in systems with abundance of prey and mates, where coalition partners are known to appropriate them in equity (such as in grassland systems of E. Africa^15,17^). However, even in such systems reproductive skew is observed within very large coalitions^18,22^. Whereas, in systems where per capita resources are scarce, lion coalitions are hierarchical and partners follow strict linear-dominance regimes (such as in the Gir forests of W. India which is primarily a woodland habitat with small modal prey and small female groups^20^). Such a linear hierarchy has been proposed to have resulted in an optimal coalition size of two male lions in the Gir system, below and beyond which the costs of coalescing (or not) are higher than the benefits for loners and subordinates^20^. However, this computation of optimal coalition size was based on fitness indices developed through behavioural metrics alone, and did not consider the potential role of genetic relatedness between partners in deciding the payoffs of cooperation.

From studies across species and systems we know that relatedness often shapes the evolution and functioning of coalitions because of two primary reasons: (1) groups fare better than solos, and (2) resource competition often make certain group members rely primarily on indirect fitness benefits (appropriated from related individuals in the group) to maintain threshold inclusive fitness levels, explained through Hamilton’s principle^1^. For example, in wild turkeys, males form display partnerships/coalitions at lekking sites, but these coalitions are always forged between related individuals. There is a hierarchy in mating opportunities within such display coalitions, and subordinates help dominants at their own costs because subordinates almost never sire any offspring but gain indirect fitness benefits by being related to the dominants who sire many^11^. Studies from bottlenose dolphins *Tursiops* spp^23,24^, Barbary macaques *Macaca sylvanus*^25^ and cheetahs *Acinonyx jubatus*^26^ have also revealed the importance of kin-selection in coalition formation. In the grassland systems of E. Africa, it has been documented that larger coalitions where reproductive skew is pronounced between partners, are always constituted of close kin^22^. Much like turkeys, in these large coalitions certain partners act as non-breeding helpers but remain as part of the coalition despite getting no/minimal chances to mate, only because they are closely related to the males that obtain majority of the mating. Kinship related benefits aid such helpers to indirectly reproduce based on shared genes^27^.

Gir lion coalitions exhibit strong reproductive skew wherein a *consistently* dominant partner appropriates > 70% of all mating events^20^. This skew is increased in coalitions with > 2 males, where the low-ranking partners barely get any chance to breed. Thus, following Hamilton’s principle^1^, we predict that such large coalitions should ideally have a higher proportion of related individuals, wherein relatedness would offset the fitness skew. A flip side to this prediction would be that unrelated males would generally coalesce when the apparent reproductive skew is not as pronounced between partners as observed within large coalitions.

We test these predictions through genetic analyses of relatedness between male partners in coalitions of varying sizes of free-ranging lions inhabiting the Gir forests of Gujarat, India. We couple our genetic analyses with extensive behavioural information on these coalitions to assess how relatedness affects male cooperation. Lions in Gir being highly inbred pose a definitive challenge for decoding relatedness^28,29^. To address this challenge we use a panel of microsatellites to genotype lions and compute coefficient of relatedness for mother–offspring pairs, siblings/littermates, and pairs that are unlikely to be related (known through long-term field records). We measure relatedness within male coalitions relative to these known individuals. Using this genetic coefficient of relatedness, we computed fitness indices that represented potential number of offspring sired by males and compared them with earlier indices that used only behavioural metrics. Subsequently, through the quantification of behaviour of individual lions during territorial confrontations compared across related and unrelated partners, we assess the influence of kin-selection on immediate cooperation.

While there have been definitive studies on the ultimate cost–benefit trade-offs for subordinates in aiding dominants, few have addressed what the dominants might gain and/or lose in tolerating subordinates in a group (reviewed in Refs.^2,30^). In lion coalitions from Gir, not all the mating events are acquired by the dominant individuals, and the subordinates get chances to breed (although minimally). However, the subordinates obtain mating opportunities only when dominant(s) are not around, and get supplanted if dominants are present^20^. Such acts by subordinates thus can be attributed to an ‘incomplete control’ or ‘failure to exert complete dominance by the alpha(s)’^30^. Consequently, mating events perpetrated by subordinate males are costs or loss of opportunities for dominant individual(s). We provide a novel examination of how relatedness between dominants and subordinates might even aid in amending such losses for the dominants, further propagating cooperative behaviour.

Finally, we combine these behavioural and genetic data with long-term demographic information through probabilistic models that estimate kin availability for coalition formation, thus constraining evolutionary optimality in real world—an infrequent occurrence in studies on cooperation. With these models we revisit the concept of optimal coalition size and show that the group size of male-alliances that yields the best possible fitness outcome is not the most prevalent in nature.

## Results

### Genetic relatedness

The 14 microsatellite markers used in the study were found to be polymorphic, with an average polymorphic information content of 0.73. The number of alleles per locus ranged from four alleles at locus PLE23 to a maximum of 12 alleles at the locus PLE57 (Supplementary Table S1). The P_ID_ and PID_ID-sib_ values for the panel of microsatellites was 8.7 × 10^–14^ and 1.3 × 10^–6^ respectively.

### Threshold coefficients of relatedness

Pairwise relatedness coefficients were estimated using Queller and Goodnight QG^31^ and TrioML^32^ estimators. While both the estimators revealed similar distributions of coefficients (Supplementary Data S1), we hereafter report relatedness coefficients based on TrioML estimator because it is known to perform better for inbred populations^32^. For details of genetic analyses, please see the Methods section. Average coefficient of relatedness between littermates and mother–offspring pairs was 0.63 with a 95% lower bound of 0.58. Average relatedness between individuals that were unlikely to be closely related was 0.36, with a 95% upper bound of 0.40 (Supplementary Data S1).

### Genetic relatedness between coalition partners

Average coefficient of relatedness between male partners in coalitions of 2 males (n = 13 individuals) was 0.43 ± 0.08, lower than partners in large coalitions of > 2 males (n = 10 individuals) having an average relatedness of 0.50 ± 0.07 (Table 1). Subsequent identification of related partners showed their higher occurrence in large coalitions (freq. = 0.67) than in pairs (freq. = 0.29) (Table 1). Every large coalition had at least 2 males that were littermates. There were four pairs of lions in three large coalitions, whose relatedness was higher than that of ‘unrelated’ individuals, but lower than the known related lions. We classified them into the half-sibling/cousin category. For details of classification, please see the Methods section.

**Table 1:** Coefficient of relatedness (measured using TrioML estimator) between male coalition partners belonging to 10 coalitions (n = 23 individuals) of free-ranging Asiatic lions, out of which behavioural information on dominance hierarchies are available for 17 males in seven coalitions that also featured in^20^.

Coalition size		Partner 1 (Rank 1)	Partner 2 (Rank 2)	Partner 3 (Rank 3)	Partner 4 (Rank 4)
2	Partner 1	–	0.22 (0)	–	–
2	Partner 1	–	0.39 (0)		
2	Partner 1	–	0.39 (0)	–	–
2	Partner 1	–	0.22 (0)	–	–
2	Partner 1	–	0.34 (0)	–	–
3	Partner 1	–	**0.5 (0.18)**	**0.88 (0.5)**	–
	Partner 2	–	–	0.31 (0)	–
4	Partner 1	–	**0.52 (0.18)**	**0.62 (0.5)**	**0.84 (0.5)**
	Partner 2	–	–	0.14 (0)	0.29 (0)
	Partner 3	–	–	–	**0.55 (0.18)**
**Male partners in the coalitions underneath are not arranged as per their dominance ranks because behavioural data was not available for these coalitions**					
2	Partner 1	–	**0.69**^**a**^** (0.5)**	–	–
2	Partner 1	–	**0.78 (0.5)**	–	–
3	Partner 1		**0.72 (0.5)**	0.18 (0)	
	Partner 2	–	–	**0.41 (0.18)**	–

These 17 males have been arranged with respect to their behavioural dominance ranks. Coefficients that represent related males are marked in bold. The numbers in parentheses beside the coefficients represent Hamiltonian relatedness coefficient between each pair. Hamiltonian relatedness coefficients correspond to 0.5 (siblings/parent-offspring), 0.18 (average value for half-sibling/cousin because we could not discriminate between them) and 0 (unrelated).

^a^Possibly denotes a father–son coalition because the age difference between the 2 males was ~ 5 years.

### Fitness indices of coalition partners

**Figure 1:**
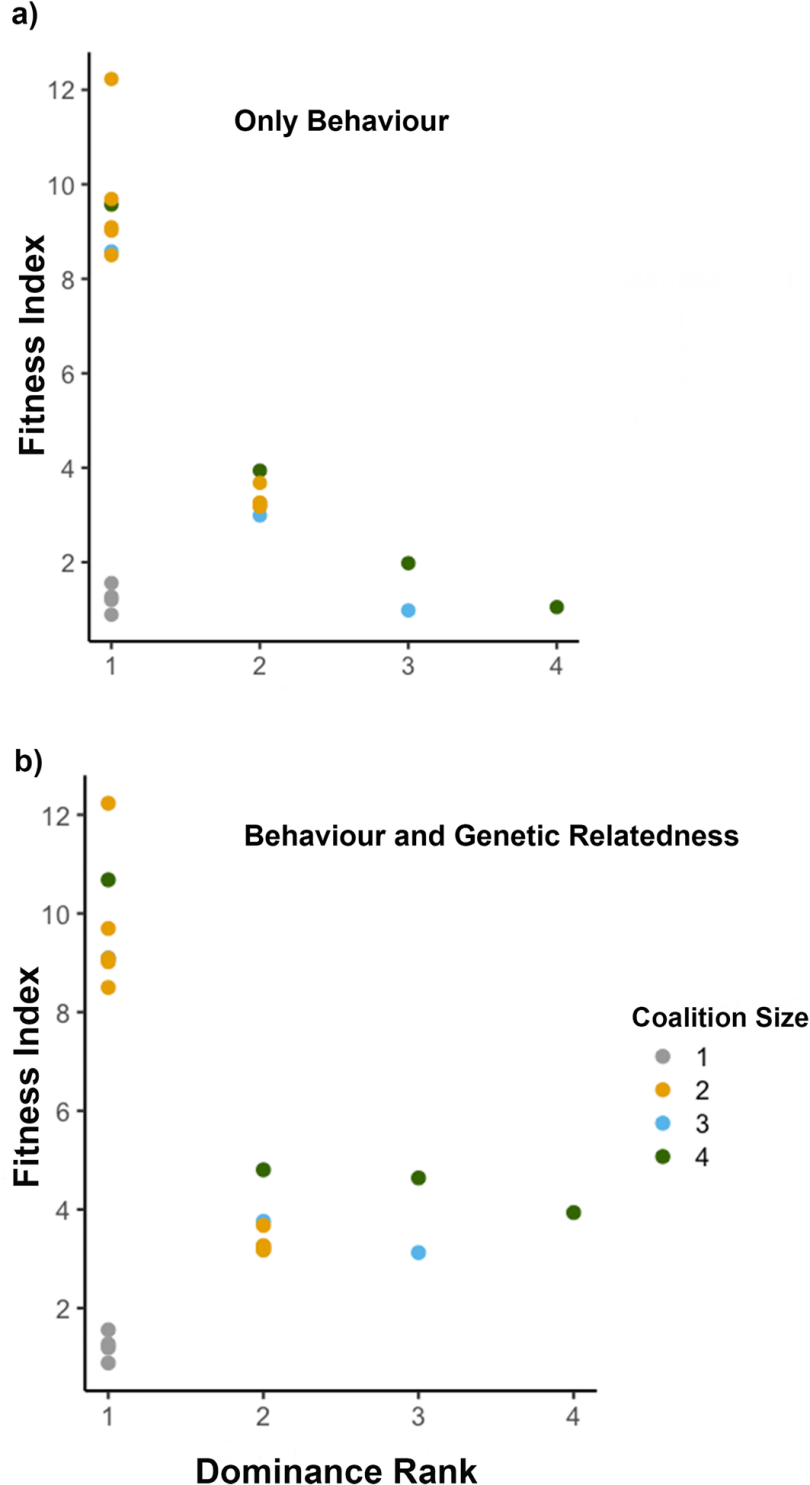
Fitness indices of individual lions belonging to different coalition sizes and behavioural ranks within their respective coalitions. (**a**) Fitness indices computed using only behavioural metrics, redrawn as is from^20^. Herein, fitness index of every male = annual territory holding probability*mating frequency, (**b**) Fitness index calculated by incorporating the genetic relatedness between the partners for every coalition, with mating frequencies and annual territory holding probabilities obtained from^20^. In this genetic based computation, for every individual the previously computed fitness index was augmented by r*mating frequency of another partner in the coalition. This augmentation was done for and between every individual in the coalition. *r* took the value of 0.25 if the partners were full-siblings, 0.09 if they were half-siblings/cousins, and 0 if they were unrelated.

Calculation of fitness indices by incorporating genetic relatedness between partners revealed that subordinates in large coalitions (> 2 males) had fitness indices higher than indices computed through behavioural observations only (Fig. 1). Low-ranking partners in large coalitions (> 2 males), although being behaviourally inferior had ultimate fitness indices comparable to subordinates of 2 male coalitions. By considering indirect effects, fitness indices of low-ranking subordinates in such coalitions increased by 196 ± 36% as compared to indices computed only through behavioural metrics. Dominant individuals lost 9% less if their mating opportunities were compromised to related subordinates than unrelated partners/rivals.

### Optimal coalition size in male lions

**Figure 2:**
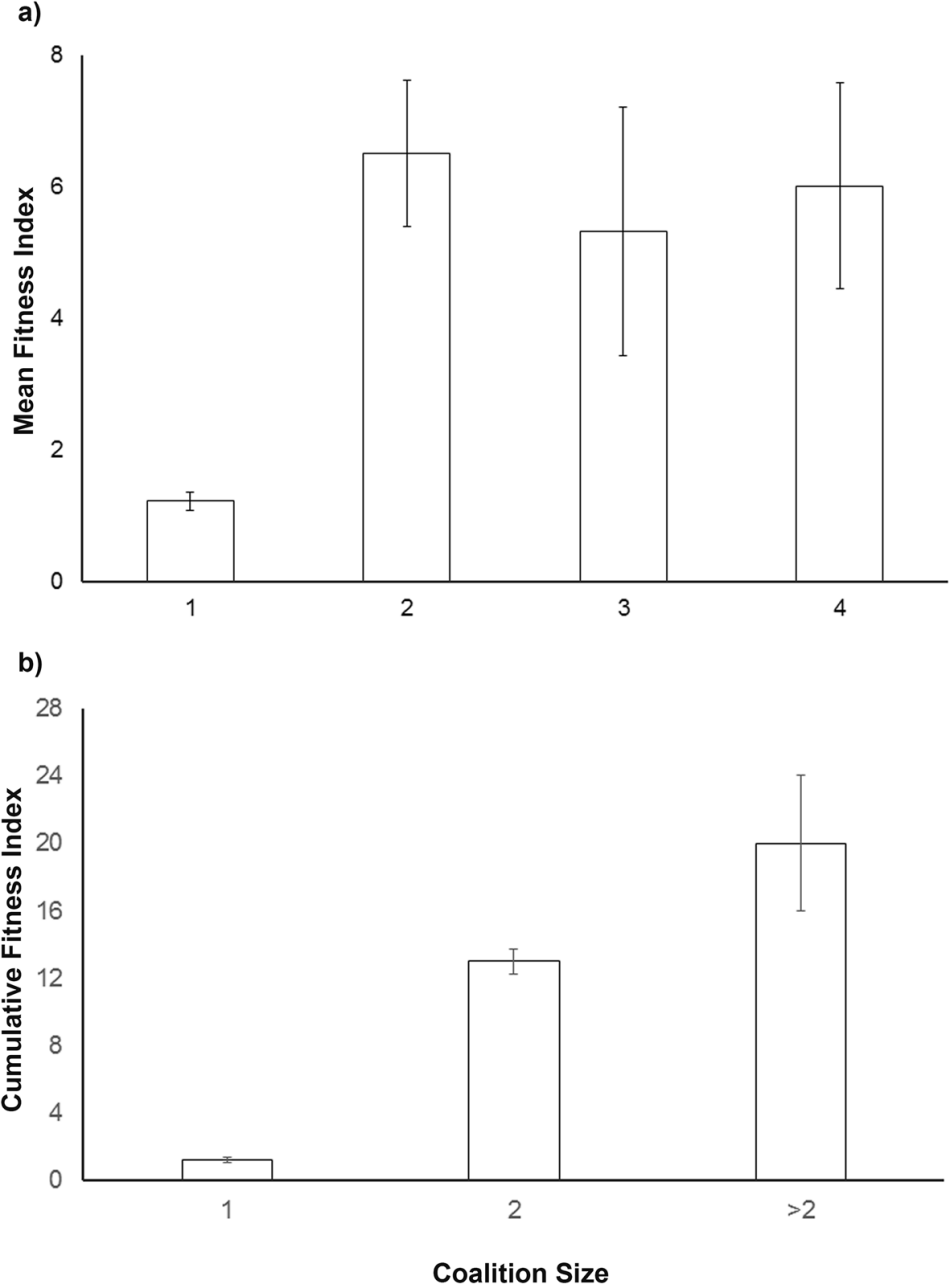
Metrics of group fitness of coalitions. (**a**) Mean fitness index: fitness index (including genetic relatedness) averaged within a coalition, (**b**) Cumulative fitness index: computed by adding the fitness indices of all the partners in a coalition. Both metrics are represented as functions of coalition size. Error bars are SEs.

The mean fitness index for individual lions was higher for males in coalitions than single males, however this fitness index that was an indicator of reproductive success was not significantly different between pairs, trios and the quartet in our study (Fig. 2a). Variance in fitness between partners was greater within large coalitions (> 2 males) than in pairs, indicating a higher disparity of per capita coalitionary benefits in large coalitions (Fig. 2a). However, the total fitness of a coalition (cumulative fitness index across partners) was higher for large coalitions (Fig. 2b).

### Demographic constraints on formation of large coalitions

The probability of two male siblings being recruited to the population was low (10%), and the ideal likelihood of large coalitions to form (considering different scenarios conditioned upon the availability of kin) was ~ 7% (please see Supplementary Notes S1 and S2 for details and computations). Thus, the observed precondition for large coalitions of having related partners seemingly acts as a limiting factor for their occurrence in the natural world. Whereas, the probability of single males being recruited to the population was substantially higher (38%). This creates relatively more opportunities for loners to either attempt holding breeding territories or to partner with unrelated males to form pairs (Supplementary Note S1), the latter being more likely because of the direct benefits to both partners in being in a coalition.

### Effect of kinship on proximate cooperation in male lions

**Figure 3:**
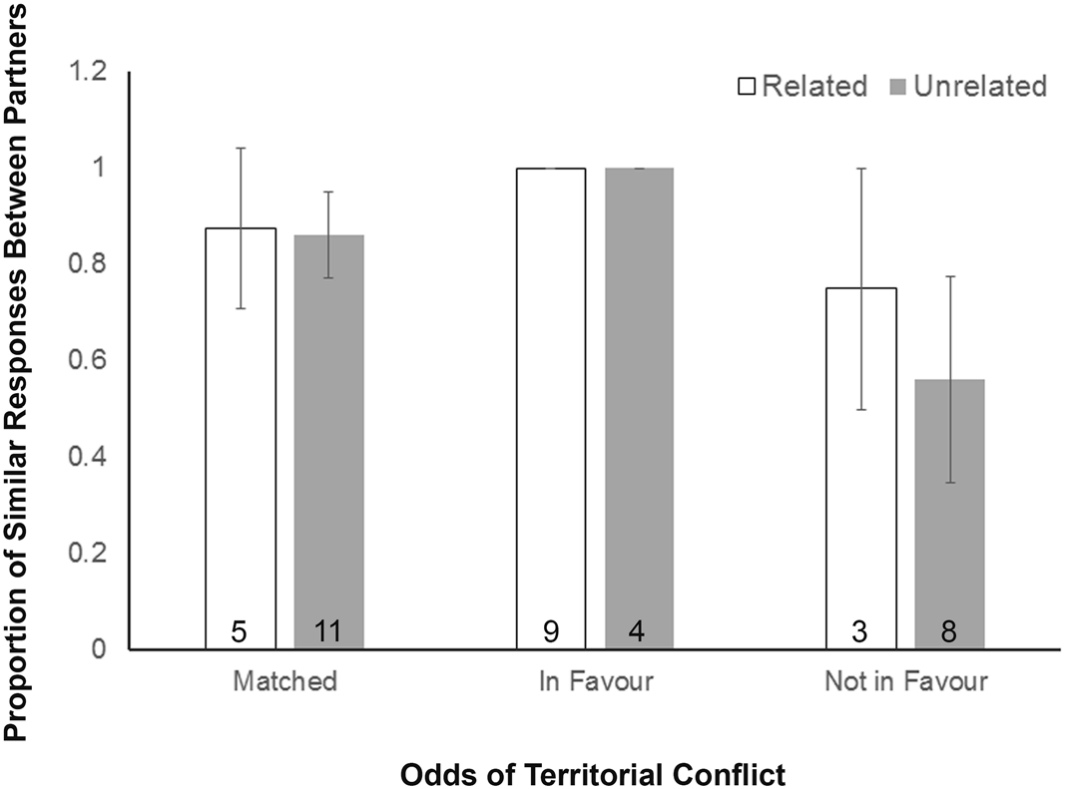
Proportion of similar responses of male coalition partners (paired interactions) during territorial conflicts. Results are compared between related and unrelated pairs for situations: (1) when the conflict odds were *matched* (opponents equally matched in numbers), (2) when odds were *in favour* (number of focal males > opposition number), and (3) when odds were *not in favour* (number of focal males < opposition number). Estimated proportions in each of the three categories represent values averaged across individual coalitions. Numbers at the base of each bar represent sample sizes. Error bars are SEs.

Decisions to cooperate or defect during territorial conflicts were not different between related and unrelated pairs (Fig. 3). Male partners were rarely found to defect/cheat (17%), and they did so typically during confrontations where they were outnumbered.

## Discussion

Our results show that small coalitions (2 males) in Gir were primarily (71%) composed of unrelated male lions, while large coalitions (> 2 males) were comprised of littermates and cousins/half-siblings. A previous study^20^ documented proximate behavioural suppression between dominant and subordinate individuals: dominants appropriated more mating and supplanted the latter from mating and feeding events. However, when we incorporated genetic relatedness between partners in indices of reproductive fitness, partners at lower behavioural ranks of even large coalitions fared similarly in fitness to that of second ranking males, and had higher fitness than territorial loners. This demonstrates the benefits of male lions being in large coalitions when partners are related, which is contrary to the previous postulate of low-ranking subordinates in large coalitions faring “equally poorly” as that of single males^20^. This pattern supports our hypothesis that the skew in reproductive fitness within large coalitions is potentially offset through indirect fitness benefits to subordinates accrued through kinship. Our results also align with studies on Assamese macaques *Macacca assamensis*^12^, chimpanzees *Pan troglodytes*^33^, and Guinea baboons *Papio papio*^34^ where close kin generally form strong bonds but cooperation is not only restricted to related individuals^35^.

In the genetic based fitness calculations, we augmented the mating frequency of a male by the mating frequency of all *related* partners in the coalition, irrespective of their mutual ranks. In this model, not only do the costs emanating from being behaviourally suppressed get ameliorated for subordinates, but the ultimate loss of opportunities for high-ranking individuals also reduce if they lose mating acts to related subordinates instead of unrelated rivals. Given this evidence, indirect fitness benefits appear to be served both ways across the dominance hierarchy in lion coalitions.

Unrelated males were observed to team up primarily to form pairs, which aligns with our predictions. In such coalitions the difference in fitness resulting from behavioural suppression between partners are the least, and this allows a subordinate to have higher fitness than a loner, even without being related to the dominant male. Cooperation between unrelated males show that perhaps kinship is not a crucial prerequisite for coalition formation in lions, however, unrelated males only team up when all partners gain considerable *direct* paybacks from the association. Kin-support becomes important for coalitions in which some partners have to rely on indirect benefits to keep the cooperation beneficial across all ranks of the hierarchy. These results mirror evidence from other systems such as in male lions in Tanzania^22^.

**Figure 4:**
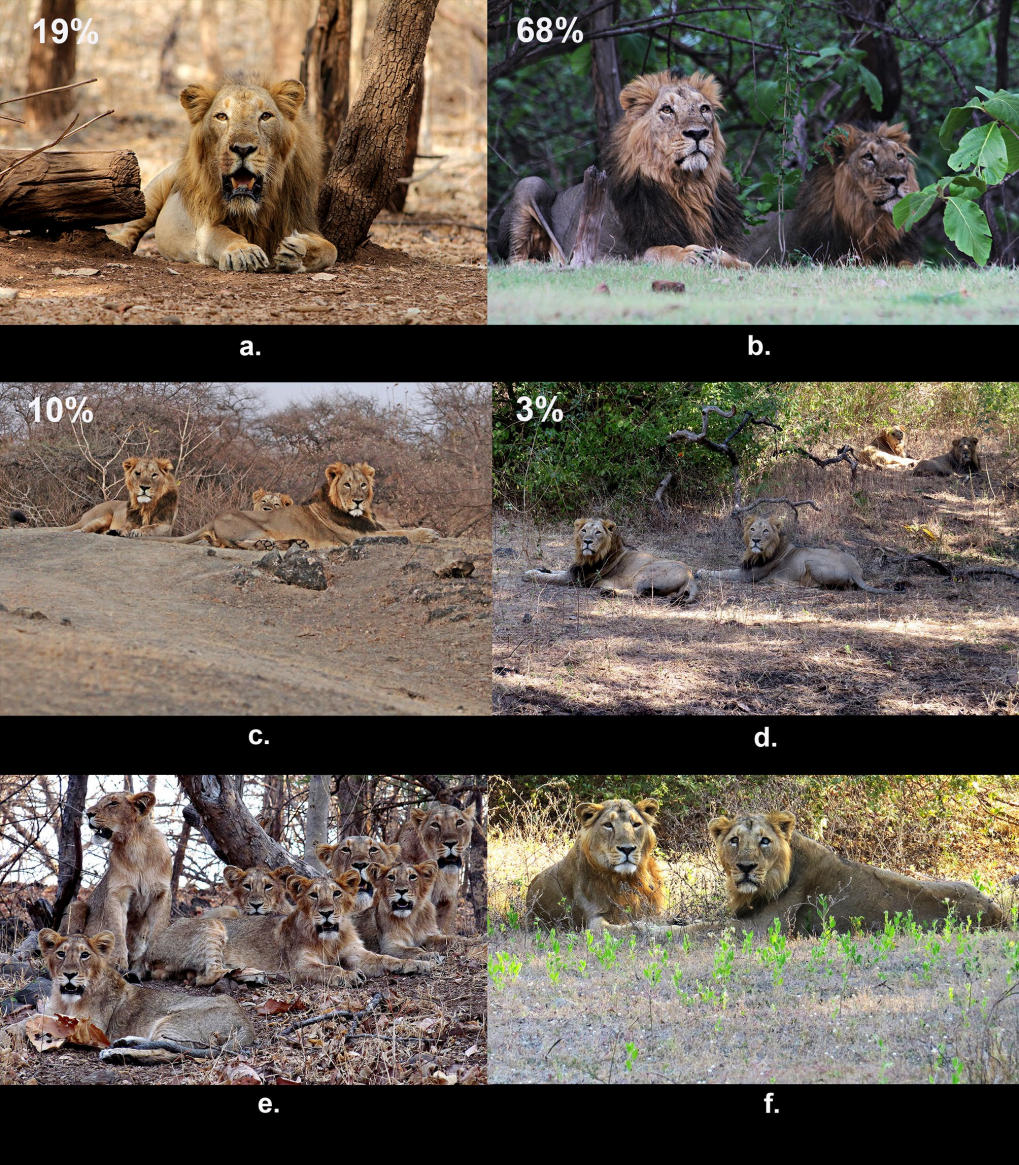
Panel of images of free-ranging Asiatic lions showing: (**a**) single male coalition, (**b**) two-male coalition, (**c**) three-male coalition, (**d**) four-male coalition, (**e**) a cohort of two synchronous litters born to a pride (mothers in the background) with 5 male juveniles (in the foreground). Such cohorts, essential in providing the initial ‘stock’ of related males to form large coalitions, are found rarely, and (**f**) a related pair where the partners are most likely father and son owing to the difference in their ages. The male to the right of the picture is ~ 10 years old, while his partner to the left is barely 4–5 years old. The percentages on the first four images represent the proportion of the respective coalition size amongst an observed pool of coalitions in Gir (n = 37). Photographs taken by Stotra Chakrabarti.

The mean fitness index of individual lions was higher for coalitions than for single males, but this mean index (a proxy for reproductive fitness) was similar for pairs and large coalitions in our study. As mentioned earlier, the magnitude of behavioural suppression in large coalitions reaches to an extent such that it appears necessary for partners to be related in order to offset this suppression through indirect fitness benefits. However, our probabilistic computations suggest that demography heavily constraints the availability of related males for facilitating colaition formation. We found that single males have higher chances of reaching dispersal age, and thus it is more likely that one would find single males and unrelated pairs in Gir, which mirrors the field-observed scenario of 68% of pairs, 19% of loners and 13% of large coalitions (n = 37 coalitions, Fig. 4). Unavailability of kin has been suggested to have promoted cooperation between unrelated individuals in chacma baboons *Papio hamadryas ursinus*^36^, humans^37^ and other primates^38^. We provide quantitative evidence to such arguments by showing that limitations on kin-availability coupled with groups/pairs faring better than solos can result in coalitions that are predominantly forged between unrelated males.

A previous study from the same system^20^ showed that the cost of sharing food increases linearly with coalition size, and this feeding skew is pronounced in Gir because modal prey size is small (Chital *Axis axis* ~ 45 kg^39^) and with more partners to share a kill, the linear hierarchical system becomes even more stringent. Incorporating this information into results from the present study, we conclude that while cumulative benefits for large coalitions are the highest, demographic and behavioural constraints limit their prevalence. Thus, ideally large coalitions with related partners would have been the most optimal selection, however, pairs emerged as the most readily attainable occurrence for lions in the Gir system.

The behavioural responses of male lions to territorial fights were similar between partners and were not influenced by their relatedness, which indicates that proximate cooperation within coalitions is not based on kinship. Partners helped each other irrespective of whether they were related or not. This is similar to conclusions drawn from an experimental study on lions in Serengeti where coalition partners showed similar responses to roar-playbacks^19^. This study suggested that acts of defection within coalitions are rare in case of territorial contests because injury to a partner(s) and loss of territory arising from acts of defection can be detrimental to group and individual fitness, and therefore necessitates cooperation in such life-threatening situations^19^. Similarly, the acts of defection where one partner chose to abandon a contest while his partner chose otherwise, although rare in our dataset, were observed for scenarios where the focal coalition was outnumbered by its opposition.

While males in a coalition typically belonged to the same age-class as their partners, it is noteworthy to mention that in one of the related pairs the age difference between the partners was ~ 5 years and could potentially be a father-son coalition (Fig. 4f). This rare conclusion was only possible with the combination of genetic data and field observations, and reveals multiple pathways for male-alliances to evolve in lions. Genetic samples from additional individuals alongwith information on their ancestry would help discern other forms of relatedness like half-siblings and cousins with certainty. However, herein we have presented the largest synchronous genetic and behavioural dataset, to our knowledge, on Asiatic lions.

Additional genetic data from coalitions and offspring coupled with further behavioural observations would also permit an assessment of whether or not mating sequence is related to paternity. Such an assessment could test expectations based on sperm competition theory^40^. For example, if evidence for insurance copulations and mate-guarding is present, we might expect that it will be more likely within unrelated than related male coalitions. Furthermore, because Asiatic lionesses are known to mate with males from different coalitions, sometimes in the same estrus^41^, cub paternity could possibly be conditioned upon factors like inter-coalition sperm competition and cryptic mate-choice from females, which in turn might determine male fitness and contribute to the mechanisms leading to male-male cooperation. While speculative, these predictions frame future research steps and emphasize the value of understanding the genetic basis of lion coalitions. Also, because we relied on proxies of male reproductive success, genetic data on cub paternity could better determine fitness benefits of coalition formation.

Since the broad elements of coalitionary behaviour of lions in Gir are similar to lions elsewhere (systems for which comparable data exists), we believe that these patterns typify male cooperation in lions in general. However, it would be worth investigating the relative effects of direct and indirect fitness components in shaping cooperation amongst male lions across regions that differ in resources from the systems known till date. This would allow to quantify resource-mediated plasticity in coalitionary behaviour, if any. While we have shown that kinship is apparently not the ‘only’ driver of cooperation, but it enhances benefits both ways across the dominance hierarchy (top-down and bottom-up) and is an essential element for large coalitions. This also shows that as long as inclusive fitness of a cooperating partner increases by being a part of a coalition beyond that of a single lion holding a breeding territory, behaviors for coalescing will be selected in nature. Such trade-offs can even be realized through cooperative acts operating beyond the mechanism of kin-selection, such as mutualism, reciprocity and/or acts explainable through game theoretic models. Additional fine-scale behavioural data from this system can tease apart whether the observed cooperation is only mutual or does it involve other mechanisms that punish cheaters and reward co-operators.

We also show that population parameters such as survival rate of cubs and litter size are crucial components that ultimately shape the availability of kin or the lack thereof, thereby affecting coalitionary dynamics. Any perturbation to these parameters (such as through anthropogenic processes like hunting or by inbreeding effects that are known to depress population rates) can have detrimental effects on lion behaviour. By combining novel genetic analysis with extensive field observations, we provide a different perspective on how male alliances in lions are shaped, and add to the array of evidence that captures group living in mammals.

## Methods

### Ethics statement

All permissions to carry out fieldwork and sample collection were obtained from the Office of the Chief Wildlife Warden (CWLW) (Permit No: 4/2007-2008 dated 24/4/2007, and WLP/C/1-8/permission/61/2015-16), under the provisions of the Wildlife Protection Act, 1972. Radio-collaring of lions was approved by the Ministry of Environment, Forests and Climate Change (MoEFCC), India (permit number: 22-7/2002 WL-I) and CWLW, Gujarat (permit number: WLP/26/B/356-61) and carried out under the supervision of veterinary officers and forest officials. The research was approved by the Training, Research, and Academic Council (TRAC) of the Wildlife Institute of India (WII) which examines the scientific rigour and bioethical considerations of the proposed techniques and protocols. We performed all methods adhering to the relevant guidelines and regulations laid out by TRAC, WII for studying wild animals. Gir lions are accustomed to people in vehicles and on foot at close proximity^20^ and behavioural observations for individuals were made only after prolonged habituation to our presence, without hindering their daily behavioural repertoires.

### Genetic and behavioural sampling

Between December 2012 and March 2019, genetic samples in the form of blood, tissue and hair were collected from 23 adult males belonging to 10 coalitions of free-ranging Asiatic lions in Gir Protected Area, Gujarat, India; out of which 17 males (in seven coalitions) had featured in a previous behavioural study^20^. The relative position of these 17 males in their respective coalitionary hierarchy were determined from direct behavioural observations, wherein mating frequencies and territory holding probabilities of individuals were computed by locating each and every male at least once in every 2 days between 2012 and 2017^20^. The dominance hierarchies were formulated based on disparity in appropriation of mating events and food from shared kills between partners^20^. In addition to these information, behavioural observations on territorial conflict between coalitions were also recorded, and we have reported the frequency and constituency of these conflicts in^41^. Genetic samples were collected from immobilized individuals when they were sedated either for radio-collaring or for treatment of wounds. In addition to this, as part of the long-term ongoing research project (reviewed in Ref.^42^), genetic samples were collected from known individuals across the entire landscape (> 12,000 km^2^). These samples were categorized into individuals that were (1) *related*—mother–offspring and sibling pairs that were known from records on regularly monitored prides, and (2) *unlikely to be closely related*—individuals that were sampled from areas that were distant geographically because lions in Gir occupy a spatial extent of > 12,000 km^2^. For this category, we also selected samples that belonged to individuals that were separated by 5–15 years with no known interactions between them. The temporal and spatial separation between these individuals was to reduce the potential of close relatedness between them. For details of the study area, selection of coalitions and prides and their monitoring schemes, see^20,41^.

### Analysis of genetic relatedness

Genomic DNA was extracted from blood, tissue and hair samples using DNeasy blood and tissue kit (QiagenValencia, CA, USA)^43^. Following extraction, DNA from these samples was processed for genotyping using a panel of 14 microsatellites selected from published research on African lions^44^, Asiatic lions^45,46^ and other felids^47–49^. Each microsatellite loci was amplified in a singleplex 12 μl reaction volume containing 3 μl of 2 × Multiplex MasterMix with HotStart Taq Polymerase (Qiagen), 2 μl of Q solution, 0.2 μm labelled forward and 0.2 μm of unlabelled reverse primer and 5 μl of extracted DNA. These reactions were set at conditions of: initial denaturation (95 °C for 15 min), 40 cycles of denaturation (94 °C for 45 s), annealing (varied between 51–58 °C for 30 s) and extension (72 °C for 45 s) and a final extension (60 °C for 30 min). These singleplex reactions were multiplexed post-PCR and processed for genotyping on an ABI 3500XL sequencer, and resultant alleles were scored visually on Geneious V5^50^. Alleles were visually identified, and binned using Tandem 1.01^51^. All reactions were performed in triplicates to reach a consensus genotype, to account for error in genotyping^52^. Pairwise relatedness coefficient was estimated using the Queller and Goodnight (QG) estimator^31^ implemented in Genalex 6^53^ and TrioML estimator^32^. TrioML estimator is known to perform better for inbred populations, and at estimating relatedness in samples with unknown pedigree^32^. Since ancestry allele information was not used to arrive at the relatedness matrix, the coefficients default to relative relatedness coefficients^32^. The absolute value of these coefficients however would depend on the microsatellite panel (number of loci, their allelic richness and their frequency in the study population) as well as the population of lions used for the computation.

### Computation of threshold coefficients to discern sibling relatedness

From long-term records we identified genetic samples of 24 individuals (Supplementary Data S1) that were either mother–offspring dyads (n = 7 pairs, 13 individuals) or full-siblings/littermates (n = 7 pairs, 11 individuals). Based on our panel of microsatellites, we calculated the average coefficient of relatedness amongst these 24 individuals and computed a 95% lower bound on this value. We considered coalition partners with relatedness coefficients higher than this lower bound as littermates/full-siblings. Similarly, we developed a 95% upper bound on the mean relatedness between individuals that were most unlikely to be related (n = 13 individuals, 78 pairs). We considered coalition partners with relatedness coefficients lower than this upper bound to be unrelated. We coupled genetic analysis with long-term genealogical records to develop threshold distributions because the population is inbred and base-relatedness between individuals is presumably high, similar to the approach adopted in Ref.^54^. However, coalition partners having relatedness coefficients lying in between the 95% upper bound of unrelated individuals and the 95% lower bound of related individuals could not be established to be completely unrelated, and for this study we considered them to be half-siblings or cousins because in lions, half-siblings and cousins are known to join in coalitionary partnerships. This is because multiple lionesses in a pride (who are matrilineally related) often give birth synchronously resulting in a crèche of cubs that have different mothers but same father (i.e. half-siblings) and/or cubs that are sired by different fathers (unrelated coalition partners) to different mothers who are matrilineally connected (i.e. cousins)^21,22^. Since both the QG and TrioML estimators resulted in similar threshold distribution and inferences, we subsequently used coefficients from the TrioML estimator that is known to perform better for inbred populations. The values of relatedness coefficients that we report would apply only to the study sample of lions as they would be dependent on the population used for computing these coefficients and the panel of microsatellites selected.

### Genetic relatedness between coalition partners

We compared the coefficient of relatedness between male partners belonging to different coalition sizes to find out how average relatedness varies with coalition size. Using threshold distributions, we also identified full-siblings, cousins and/or half-siblings within coalitions, and compared the frequency of occurrence of such related partners between small and large coalitions.

### Fitness index of coalition partners

Current understanding of coalitionary hierarchy in this system is based on an index that surrogates for individual direct fitness (*fitness index* = mating frequency * annual territory holding probability) of male lions^20^. This index surrogates for the number of offspring potentially sired by a male across its reproductive/territorial tenure, because males are known to sire cubs only when they are territorial^21^. Mating/copulation frequency is known to be fairly accurate in predicting parentage in certain species such as lions^55^, red deers *Cervus elephas*^56^ and mandrills *Mandrillus sphinx*^57^, however such frequency does not always match with the number of offspring sired. But evidence suggests that males who appropriate more matings typically sire more offspring than males who mate less^11^. Thus, in the absence of paternity data, which is often difficult to collect in natural systems and more so in our system where non-invasive genetic sampling of cubs was constrained by sample sizes, this index can act as a reasonable proxy of reproductive success when used on a ‘comparative scale’ between males. Comparison of this index between coalition partners has established the linear hierarchical system within Asiatic lion coalitions. In linearly hierarchical social systems, the most dominant partner is at the pinnacle of lifetime success, while the success of any subordinate is relative to that benchmark as well to the success of the immediate dominant individual(s)^58^. In such hierarchical groups, the decision for any subordinate to stay or leave the group depends upon the loss of opportunities it incurs relative to all the dominant individuals, as well as what it is likely to achieve if it were to attempt holding a territory on its own. It is the difference between these parameters that determines whether an individual should continue being a subordinate in a coalition or leave and be a loner. Comparison of fitness indices between coalition partners is based on the fact that mating frequency is different between the partners, with subordinates getting disproportionately fewer mating than the dominant individual(s)^20^. This loss in opportunity to mate is the primary cost of coalescing for subordinates, because fewer matings would ideally result in less chances of propagating their own genes, thereby reducing their inclusive fitness. However, if subordinates are related to the dominant individual(s), then such loss of opportunities (mating events) can be augmented through indirect benefits. To simplify, if a dominant partner (*i*) has a mating frequency of X*i* and the subordinate (*j*) has a mating frequency of X*j* (where X*i* > X*j*), and the males are related, assuming both have equal probability of fertilization when they mate with any female; then ultimately the reproductive success of the subordinate would be:

X*j* + *r*_ij_*Xi (*r*_*ij*_ = 0.25 if the males are full-siblings and *r*_*ij*_ = 0.09 if the males are half-siblings or cousins).

This is because, every cub sired by partner (*i*) from those mating events would be related to a subordinate full-brother by 0.25 or to a subordinate half-brother by 0.125 or to a subordinate cousin (*j*) by a coefficient of 0.06^1^. Since we had no way of confirming whether a male was a half-sibling versus a cousin, we used the average value of *r* = 0.09 for probable half-sibling (0.125) and/or cousin (0.06). Considering there are multiple partners in a linearly-hierarchical coalition with varying degrees of relatedness, this augmentation of apparent reproductive success for a subordinate should occur from every individual it is related and subordinate to. Thus, considering indirect fitness benefits, the reproductive success of the individual (*j*) at the bottommost rank of a coalition of 3 males (*h,i,j*)would be:

$${\text{X}}j + r_{ij} *{\text{X}}i + r_{hj} *{\text{X}}h.$$

This follows calculations derived from the theory of kin-selection where relatedness between individuals lead to shared inheritance^1, 59^. While these calculations are based on what the subordinate(s) might gain by being related to the dominant(s), there are also definitive costs for dominant individual(s) to share the same territory with subordinate(s). The major cost is mating events appropriated by subordinate males when the former is not around. However, the physical absence of the dominant individual from the vicinity of lioness(es) in estrus that results in such lack of mating appropriation might have anyway resulted in him losing these opportunities (if not to his coalition partner but to rival coalitions, because lionesses in our study system often mate with multiple rival coalitions^41^). We hypothesize that losing such opportunities to related partners would be beneficial for dominant males than getting compromised to unrelated rivals. Following this theory, we supplemented the mating frequency of the dominant individual(s) by the mating events appropriated by related subordinates (similar to the aforementioned fitness calculations that we used for augmenting the apparent reproductive success of subordinates). We considered this supplementation as a plausible mechanism that would ultimately amend the loss of mating opportunities that high-ranking individuals might experience, thus benefitting the related dominant(s) as well. Mating frequency values correspond to an annual rate = (no. of mating events appropriated by a male/no. of days detected in field)*365^20^.

### Optimal coalition size in male lions

Coalition males should ideally cooperate to maximise the fitness of the group as a whole, while each member of the coalition should strive to maximise its individual fitness; resulting in strife between cooperating partners. These conflicting mechanisms modulated by demographic processes that dictate availability of partners determine coalition sizes observed in the natural world. We measured individual fitness, mean group fitness, variability of fitness in coalitions, and cumulative group fitness to evaluate optimal coalition size. To these parameters we qualitatively integrated published information on feeding skew (difference in prey biomass consumed between coalition partners from shared kills^20^) as a primary cost of sharing resources in a coalition (in addition to the reproductive skew). Published information reveals that with unit increase in coalition size, the feeding skew between partners increases linearly by a factor of 0.31, considering an average prey size across these feeding events^20^. We discuss a conceptual cost–benefit trade-off across different coalition sizes considering reproduction and food intake as the two primary parameters of interest.

### Demographic constraints on coalition formation

Though selection would strive to reach optimality, it would be constrained in the real world by availability. Herein we used demographic parameters of lions based on ~ 20 years of data on this population, such as juvenile survival rate and litter size^60^, and information on synchronous litters^41^ to model the probabilistic processes that produce males of appropriate age (2–3 years, when young males get ousted from their natal prides and enter a stage of nomadism and form coalitions) within a pride for the formation of coalitions (please see Supplementary Notes S1 and S2 for details). We used these computed probabilities to create scenarios of large coalitions to exist in the wild, conditioned upon the information on occurrence of related partners in such coalitions. Such probabilistic calculations were pursued to understand how the availability of kin (or the lack thereof) affects coalition formation in lions. Also, by using information on group sizes of male lions (n = 37 coalitions) observed across the Gir landscape^20,41,61–63^ we checked whether our models align with ground reality.

### Effect of kinship on proximate cooperation in male lions

To investigate the effect of relatedness between coalition partners on proximate acts of cooperation, we used information on territorial conflicts between coalitions as reported previously (n = 28; Ref.^41^). Male coalitions are known to work as a cohesive unit while defending their territories against intruders, and while invading into foreign territories^15,19^. We checked the behavioural responses of individual male partners to such acts of defence or intrusion, hypothesizing that if cooperation was based on kinship alone then responses of related males to such conflict scenarios would be similar to each other more often than for unrelated males. Unrelated partners should defect/cheat more frequently as compared to related males when presented with life-threatening situations such as territorial conflicts. We did not consider events where just single males were involved on both sides. For every conflict event, we did a pairwise comparison between coalition partners (n = 40 paired interactions, Supplementary Data S2) and checked if both the partners had similar simultaneous response (fight or flight) or one of them abandoned while the other chose to fight/stand its ground. We compared related versus unrelated pairs by presenting this information as proportions of similar responses between partners across three different scenarios: (1) when the conflict odds were matched (opponents equally matched in numbers), (2) when odds were in favour (number of focal males > opposition number), and (3) when odds were not in favour (number of focal males < opposition number). Estimated proportions in each of the three categories represent values averaged across individual coalitions.

All data processing was done using MS Excel and analyses using program Rv3.6.1^64^. Errors are SEs if not specified otherwise.

### Supplementary information

Supplementary file1

Supplementary file2

Supplementary file3

Supplementary file4

Supplementary file5

Supplementary file6

## Data Availability

All data generated or processed during this study is available either in the Supplementary Information or as published sources. Reference to the respective published sources are in the Main Text.

## Glossary of terms

Actor: Focal individual performing a behaviour.
Benefits: Opportunities to gain direct and/or indirect fitness.
Costs: Loss of opportunities to gain direct and/or indirect fitness.
Direct fitness: Fitness gained from producing offspring; the component of personal fitness ascribed to one's own/independent behaviour.
Indirect fitness: Fitness gained from aiding related individuals/kin.
Inclusive Fitness: Sum of direct and indirect fitness components of an individual.
Kin-selection: A process through which certain traits are selected for because of their positive effect(s) on the fitness of related individuals.
Mutualism: 2-way cooperation between individuals.
Recipient: An individual affected by the behavioural act(s) performed by a focal individual (actor).
