# Author Correction: The role of kinship and demography in shaping cooperation amongst male lions

**DOI:** 10.1038/s41598-024-54226-2

**Published:** 2024-02-14

**Authors:** Stotra Chakrabarti, Vishnupriya Kolipakam, Joseph K. Bump, Yadvendradev V. Jhala

**Affiliations:** 1 Department of Fisheries, Wildlife & Conservation Biology, University of Minnesota, 2003 Buford Circle, 150 Skok Hall, St. Paul, MN 55108 USA; 2 Department of Animal Ecology & Conservation Biology, Wildlife Institute of India, Chandrabani, Dehradun, Uttarakhand 248 001 India

Correction to: *Scientific Reports* 10.1038/s41598-020-74247-x, published online 16 October 2020

The original version of this Article contained an error. In the Methods, under the subheading ‘Ethics statement’, the permit number of the Office of the Chief Wildlife Warden (CWLW) was incorrect.

As a result,

“All permissions to carry out field work and sample collection were obtained from the Office of the Chief Wildlife Warden (CWLW), Gujarat under the provisions of the Wildlife Protection Act, 1972 (permit number: WLP/28/C/97-99/2011-16). Radio-collaring of lions was approved by the Ministry of Environment, Forests and Climate Change (MoEFCC), India (permit number: 22-7/2002 WL-I) and CWLW, Gujarat (permit number: WLP/26/B/356-61), and carried out under the supervision of veterinary officers.”

now reads:

“All permissions to carry out fieldwork and sample collection were obtained from the Office of the Chief Wildlife Warden (CWLW) (Permit No: 4/2007-2008 dated 24/4/2007, and WLP/C/1-8/permission/61/2015-16), under the provisions of the Wildlife Protection Act, 1972. Radio-collaring of lions was approved by the Ministry of Environment, Forests and Climate Change (MoEFCC), India (permit number: 22-7/2002 WL-I) and CWLW, Gujarat (permit number: WLP/26/B/356-61) and carried out under the supervision of veterinary officers and forest officials.”

The original Article has been corrected.

