# Supplementary material for: The role of kinship and demography in shaping cooperation amongst male lions
Source: Sci Rep. 2020 Oct 16;10:17527. doi: 10.1038/s41598-020-74247-x (PMC7568578; doi:10.1038/s41598-020-74247-x)
Supplement: Supplementary file 3 — Supplementary file3 [file 41598_2020_74247_MOESM3_ESM.pdf]

Manuscript: THE ROLE OF KINSHIP AND DEMOGRAPHY IN SHAPING COOPERATION AMONGST MALE LIONS

Authors: A) Stotra Chakrabarti, **B)** Vishnupriya Kolipakam, **C)** Joseph K. Bump, **D)** Yadvendradev V. Jhala

Supplementary Note 1: Demographic constraints on the formation of large coalitions

Published literature from African lion systems show that large coalitions are primarily made of brothers, and partners in such coalitions are (thus) very equally matched in age [21]. Genetic analysis of coalition partners in the present study revealed a similar trend with large coalitions (>2 males) exhibiting a high likelihood of having at least two closely related partners. From the fitness analysis, it is evident that the behavioural suppression of low ranking males in large coalitions is typically ameliorated through kin-selected benefits, a crucial necessity for large coalitions to operate. Although group fitness was found to be the highest for large coalitions, such coalitions are rare in the natural system. To understand if the availability of related partners limits the prevalence of large coalitions, we modelled different scenarios where such coalitions could exist in the wild using the following population parameters of Asiatic lions:

a) Probability of a new-born being male (pM) = 0.5 (same as it being a female, pF) because litter sex-ratio does not significantly depart from 0.5 [41]

b) Survival probability of a new-born to reach dispersal age (pS) = 0.51 [60]

c) Probability of litter size being 1 (p1), 2 (p2), 3 (p3) and 4 (p4) = 0.13, 0.51, 0.29 and 0.07 respectively [60].

d) Probability of at least 2 synchronous litters (litters separated in time by a maximum of 6 months) been born as a cohort = 0.31 [41] (we used 6 months as the maximum temporal

separator because in large coalitions observed in our long-term dataset, the partners were typically very equally matched in age, with hardly any discernible differences).

The computations underneath provide information on the probability of males reaching coalitionary/dispersal age (2-3 years, when they leave their natal pride and enter into a period of nomadism and form coalitions), because this parameter is the most crucial pre-requisite for coalitions to be observed in nature.

Initial Computations:

$$\mathrm{P} = \frac{n!}{x!\,(n-x)!} \times p^{x} \times q^{(n-x)}$$

P = the probability that the unordered outcome will occur

n = total number of events
x = number of events in one category (e.g., males)
p = individual probability of *x*
q = individual probability of the other category (eg., females)

Calculation of probability of male and female cubs in different litters considering litter sex-ratio = 0.5

- **Litter size is 1:**

Probability of 1 male = Probability of 1 female =0.5

- **Litter size is 2:**

Probability of 2 males, 0 females

$$\mathrm{p} = \frac{2!}{2!\,(2-2)!} \times 0.5^{2} \times 0.5^{(2-2)} = 0.25$$

Probability of 1 male, 1 female

$$\mathrm{p} = \frac{2!}{1!\,(2-1)!} \times 0.5^{1} \times 0.5^{(2-1)} = 0.5$$

Probability of 0 male, 2 females

$$\mathrm{p} = \frac{2!}{0!\,(2-0)!} \times 0.5^{0} \times 0.5^{(2-0)} = 0.25$$

- **Litter size is 3:**

Probability of 3 males, 0 females

$$p = \frac{3!}{3!\,(3-3)!} \times 0.5^3 \times 0.5^{(3-3)} = 0.125$$

Probability of 2 males, 1 female

$$p = \frac{3!}{2!\,(3-2)!} \times 0.5^2 \times 0.5^{(3-2)} = 0.375$$

Probability of 1 male, 2 females

$$p = \frac{3!}{1!\,(3-1)!} \times 0.5^1 \times 0.5^{(3-1)} = 0.375$$

Probability of 0 male, 3 females

$$p = \frac{3!}{0!\,(3-0)!} \times 0.5^0 \times 0.5^{(3-0)} = 0.125$$

- **Litter size is 4:**

Probability of 4 males, 0 females

$$p = \frac{4!}{4!\,(4-4)!} \times 0.5^4 \times 0.5^{(4-0)} = 0.0625$$

Probability of 3 males, 1 female

$$p = \frac{4!}{3!\,(4-3)!} \times 0.5^3 \times 0.5^{(4-3)} = 0.25$$

Probability of 2 males, 2 females

$$p = \frac{4!}{2!\,(4-2)!} \times 0.5^2 \times 0.5^{(4-2)} = 0.375$$

Probability of 1 male, 3 females

$$p = \frac{4!}{1!\,(4-1)!} \times 0.5^1 \times 0.5^{(4-1)} = 0.25$$

Probability of 0 male, 4 females

$$p = \frac{4!}{0!\,(4-0)!} \times 0.5^0 \times 0.5^{(4-0)} = 0.0625$$

Calculation of survival probabilities of different male cubs in litters, following the similar approach of binomial functions as used for M/F ratio in litters:

Survival scenarios (no.of male cubs|no. of survivors)	Binomial Prob.
(1|1)	0.51
(2|1)	0.499
(3|1)	0.367
(4|1)	0.24
(2|2)	0.26
(3|2)	0.382
(3|3)	0.133
(4|3)	0.259
(4|2)	0.375
(4|4)	0.068

i) The probability of one male cub in a litter surviving to dispersal age is the following joint probability of:

litter size is one and the cub is a male and it survives + litter size is two and both are males and one survives + litter size is two and one is a male and it survives+ litter size is three and all three are males and only one survives + litter size is three and two are males and one survives + litter size is three and one is a male and it survives + litter size is four and all four are males and one survives + litter size is four and three are males and one survives + litter size is four and two are males and one survives + litter size is four and one is a male and it survives, i.e

0.38

ii) The probability of two male littermates surviving to dispersal age is the joint probability of:

litter size is two and both are males and both survive + litter size is three and two are males and both survive + litter size is three and all three are males and only two survive + litter size is four and all four are males and two survive + litter size is four and three are males and two survive till dispersal age + litter size is four and two are males and both survive till dispersal age, i.e.

0.09

iii) The probability of three male littermates surviving to dispersal age is the joint probability of: *litter size is three and all three are males and all of them survive + litter size is four and all four are males and three survive + litter size is four and three are males and all three survive*, i.e.

0.007

iv) The likelihood of four male littermates surviving to dispersal age is:

litter size is four and all four are males and all of them survive, i.e.

0.0003

Ideal scenarios of large coalitions to exist considering that at least 2 partners would be related as siblings (following genetic analysis of observed coalitions):

For the sake of ideal probability, we have not considered parameters such as probability of related partners finding unrelated male(s) in the nomadic phase, or the probability of they being compatible to join

1. Two brothers team up with an unrelated third to form a trio. The likelihood of this scenario to exist is:

Probability of 2 male littermates growing up to dispersal age * Probability of one male growing up to dispersal age from a different pride (because these would likely be independent scenarios) = 0.09*0.38 = **0.034**

2. Two brothers team up with a cousin (male from a synchronized litter of the same pride) to form a trio = Probability of 2 male littermates growing up to dispersal age * Probability of one male growing up to dispersal age from another litter * Probability of 2 synchronous litters in the same pride = 0.09*0.38*0.31 = **0.010**

3. Three brothers form a trio = Probability of 3 male littermates growing up to dispersal age = **0.007**

4. Three brothers team up with an unrelated fourth to form a quartet:

Probability of three brothers surviving till dispersal age * Probability of one male growing up to dispersal age from a different pride = 0.007*0.38 = **0.003**

5. Two brothers team up with two cousins (that are brothers) to form a quartet: Probability of 2 males growing to dispersal age in one litter * Probability of 2 males growing to dispersal age in another litter * Probability of these two litters occurring in synchrony in the same pride = 0.09*0.09*0.31 = **0.002**

6. Two brothers team up with two unrelated males to form a quartet: Probability of 2 males growing to dispersal age in one litter * Probability of one male growing up to dispersal age from a different pride * Probability of one male growing up to dispersal age from yet another pride = 0.09*0.38*0.38 = **0.0130**

7. Three brothers team up with a cousin to form a quartet = Probability of three brothers surviving till dispersal age * Probability of one male growing up to dispersal age from another litter of the same pride * Probability of 2 synchronous litters occurring in the same pride = 0.007*0.38*0.31 = **0.0008**

8. 4 brothers team up = Probability of 4 male littermates surviving till dispersal age = **0.0003**

Thus, the *ideal* cumulative probability of large coalitions to form in the wild =

0.034+0.010+0.007+0.003+0.002+0.012+0.001+0.0003 = **0.071**
