# Supplementary material for: The role of kinship and demography in shaping cooperation amongst male lions
Source: Sci Rep. 2020 Oct 16;10:17527. doi: 10.1038/s41598-020-74247-x (PMC7568578; doi:10.1038/s41598-020-74247-x)
Supplement: Supplementary file 5 — Supplementary file5 [file 41598_2020_74247_MOESM5_ESM.pdf]

Manuscript: THE ROLE OF KINSHIP AND DEMOGRAPHY IN SHAPING COOPERATION AMONGST MALE LIONS

Authors: A) Stotra Chakrabarti, **B)** Vishnupriya Kolipakam, **C)** Joseph K. Bump, **D)** Yadvendradev V. Jhala

Supplementary Table S1. Diversity statistics of the panel of microsatellites used in this study, depicting number of alleles (k), observed heterozygosity (H_{obs}), expected heterozygosity (H_{exp}) and polymorphic information content (P_{IC}) of Asiatic lions.

Locus	k	H_{obs}	H_{exp}	(PIC)
FCA304	11	0.53	0.84	0.81
FCA126	9	0.70	0.72	0.67
F85	10	0.49	0.84	0.80
PLE57	12	0.73	0.83	0.82
PLE21	6	0.49	0.64	0.59
PLE23	4	0.73	0.70	0.64
PLE86	10	0.59	0.84	0.80
PLE56	9	0.65	0.77	0.72
FCA077	9	0.68	0.85	0.83
6HDZ700	11	0.67	0.85	0.82
PLE65	6	0.52	0.67	0.62
FCA441	10	0.59	0.68	0.65
FCA008	11	0.56	0.82	0.79
E7	8	0.84	0.91	0.67
**Average**	**9**	**0.63**	**0.78**	**0.73**
