# Supplementary material for: The role of kinship and demography in shaping cooperation amongst male lions
Source: Sci Rep. 2020 Oct 16;10:17527. doi: 10.1038/s41598-020-74247-x (PMC7568578; doi:10.1038/s41598-020-74247-x)
Supplement: Supplementary file 6 — Supplementary file6 [file 41598_2020_74247_MOESM6_ESM.docx]

**XI. Details of supplementary information**

**Supplementary Table S1.** Diversity statistics of the panel of microsatellites used in this study, depicting number of alleles (k), observed heterozygosity (H_obs_), expected heterozygosity (H_exp_) and polymorphic information content (P_IC_) of Asiatic lions.

**Supplementary Note 1:** Details and calculations pertaining to scenarios to understand the demographic constraints on the formation of large coalitions.

**Supplementary Note 2:** Excel-worksheet for calculations pertaining to demographic constraints on the formation of large coalitions.

**Supplementary Data 1:** Data on coefficient of relatedness between - **i)** Sheet 1: mother-offspring dyads (n=7 pairs, 13 individuals), **ii)** Sheet 2: full-siblings (n= 7 pairs, 11 individuals), and **iii)** Sheet 3: individuals that are most unlikely to be related (n=13, 78 pairs), iv) Sheet 4: male coalitions. Relatedness coefficients represent values computed using TrioML and QG estimators.

**Supplementary Data 2:** Data on behavioural outcomes from territorial conflicts (n=28) between coalitions. Data comprises of pairwise comparisons (n=40) between related and unrelated partners to territorial conflicts.
